# 4-[(3-Meth­oxy­anilino)methyl­idene]-2-phenyl-1,3-oxazol-5(4*H*)-one

**DOI:** 10.1107/S1600536812008276

**Published:** 2012-03-10

**Authors:** Wan-Yun Huang, Ye Zhang, Kun Hu, Qing-Mei Lin, Xian-Xian Liu

**Affiliations:** a Department of Chemistry, Guilin Normal College, Xinyi Road 21, Guilin 541001, People’s Republic of China; b College of Chemistry and Chemical Engineering, Guangxi Normal University, Yucai Road 15, Guilin 541004, People’s Republic of China

## Abstract

In the title compound, C_17_H_14_N_2_O_3_, the oxazolone ring is essentially planar [maximum deviation = 0.004 (1) Å] and is oriented with respect to the phenyl and benzene rings at 10.06 (9) and 5.63 (8)°, respectively; the dihedral angle between the phenyl ring and the benzene ring is 15.69 (8)°. In the crystal, N—H⋯O hydrogen bonds link the mol­ecules into chains running along the *a* axis. Neighbouring chains are inter­connected by π–π stacking, the centroid–centroid distance being 3.6201 (9) Å.

## Related literature

For background to the oxazolones, see: Fisk *et al.* (2007 ▶); Mosey *et al.* (2008 ▶); Hewlett *et al.* (2009 ▶). For the bioactivities of 4-(amino­methyl­ene)-2-phenyl-4*H*-oxazol-5-one derivatives, see: Tandon *et al.* (2004 ▶); John *et al.* (2008 ▶). For the synthesis, see: Matos *et al.* (2003 ▶). For related structures, see: Romeiro *et al.* (2010 ▶); Vasuki *et al.* (2002 ▶).
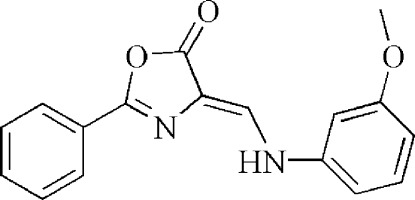


## Experimental

### 

#### Crystal data

C_17_H_14_N_2_O_3_
*M* *_r_* = 294.30
Triclinic, 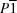

*a* = 6.6085 (5) Å
*b* = 7.1887 (5) Å
*c* = 15.3659 (10) Å
α = 98.629 (5)°
β = 94.096 (5)°
γ = 108.715 (6)°
*V* = 677.96 (8) Å^3^
*Z* = 2
Mo *K*α radiation
μ = 0.10 mm^−1^
*T* = 150 K
0.25 × 0.20 × 0.15 mm

#### Data collection

Agilent SuperNova (single source at offset) Eos diffractometer
Absorption correction: multi-scan (*CrysAlis PRO*; Agilent, 2011 ▶) *T*_min_ = 0.975, *T*_max_ = 0.985
5557 measured reflections
2759 independent reflections
2317 reflections with *I* > 2σ(*I*)
*R*_int_ = 0.023
Standard reflections: 0

#### Refinement

*R*[*F* ^2^ > 2σ(*F* ^2^)] = 0.043
*wR*(*F* ^2^) = 0.118
*S* = 1.02
2759 reflections
204 parameters
H atoms treated by a mixture of independent and constrained refinement
Δρ_max_ = 0.22 e Å^−3^
Δρ_min_ = −0.29 e Å^−3^

### 

Data collection: *CrysAlis PRO* (Agilent, 2011 ▶); cell refinement: *CrysAlis PRO*; data reduction: *CrysAlis PRO*; program(s) used to solve structure: *SHELXS97* (Sheldrick, 2008 ▶); program(s) used to refine structure: *SHELXL97* (Sheldrick, 2008 ▶); molecular graphics: *DIAMOND* (Brandenburg, 2006 ▶); software used to prepare material for publication: *SHELXL97*.

## Supplementary Material

Crystal structure: contains datablock(s) global, I. DOI: 10.1107/S1600536812008276/xu5464sup1.cif

Structure factors: contains datablock(s) I. DOI: 10.1107/S1600536812008276/xu5464Isup2.hkl

Supplementary material file. DOI: 10.1107/S1600536812008276/xu5464Isup3.cml

Additional supplementary materials:  crystallographic information; 3D view; checkCIF report

## Figures and Tables

**Table 1:** Hydrogen-bond geometry (Å, °)

*D*—H⋯*A*	*D*—H	H⋯*A*	*D*⋯*A*	*D*—H⋯*A*
N2—H2*A*⋯O1^i^	0.92 (2)	2.26 (2)	3.0110 (18)	138.1 (17)

Symmetry code: (i) 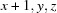.

**Fig. 1.**
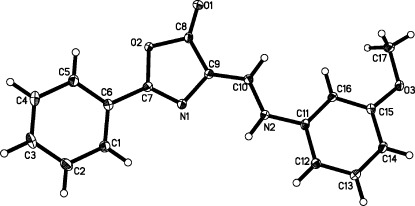
Molecular structure of the title compound showing the atomic labeling scheme with displacement ellipsoids drawn at the 30% probability level.

**Fig. 2.**
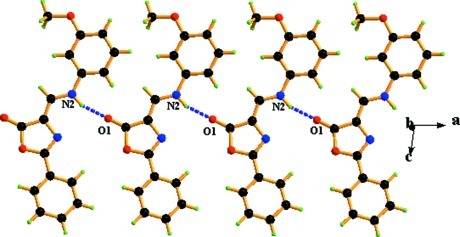
A view of the one-dimensional chain along a axis sustained by N(2)—H(2 A)···O(1)(i) ((i) x + 1, y, z) hydrogen bond (shown as dashed lines).

**Fig. 3.**
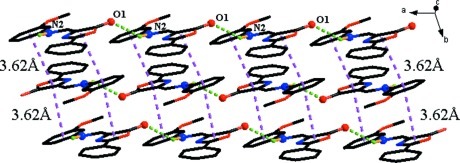
View of the two-dimensional network constructed by hydrogen bond and π-π interactions shown as green and purple dashed lines respectively.

## supplementary crystallographic information

### Comment

Oxazolones are heterocyclic compounds which serve as a very important role in the synthesis of amino acids, peptides and natural product (Fisk *et al.*, 2007; Mosey *et al.*, 2008; Hewlett *et al.*, 2009). Among them, 4- (aminomethylene)-2-phenyl-4*H*-oxazol-5-one derivatives show a range of interesting and medicinally relevant bioactivities (Tandon *et al.*, 2004; John *et al.*, 2008). Recently, Romeiro reported the crystal structure of the 4-[(Dimethylamino)methylidene]-2- (4-nitrophenyl)-1,3-oxazol-5(4*H*)-one (Romeiro *et al.*, 2010). Herein, we wish to report the synthesis and crystal structure of 4-[(3-Methoxy-phenylamino)-methylene]-2- phenyl-4*H*-oxazol-5-one. The molecule with the *Z*-configuration (Fig. 1) of the title compound is planar with the maximum deviations from the least-squares plane through all non-hydrogen atoms being 0.292 Å for atom C4 and -0.237 Å for atom C1; the r.m.s. = 0.089 Å. The sequence of C7—N1, N1—C9, C9—C10, C10—N2, and N2—C11 bond distances of 1.2917 (19), 1.407 (2), 1.372 (2), 1.337 (2), and 1.4201 (19) Å, respectively, indicate substantial delocalization of π-electron density over these atoms. The geometric parameters match closely those related structure (Romeiro *et al.*, 2010; Vasuki *et al.*, 2002). The crystal packing is dominated by N—H···O and π-π interactions. An intermolecular N(2)—H(2 A)···O(1) (Symmetry code: *x* + 1, *y*, *z*) hydrogen bond (Table 1) link the molecule into a one-dimensional chain along the *a* axis (Fig. 2). And the neighbouring chains are interconnected by π-π stacking interactions occurring between oxazolin-5-one and the 3-methoxy-phenyl ring with a centroid-centroid distance of 3.62 Å, which lead to form a two-dimensional network (Fig. 3).

### Experimental

A mixture of 4-ethoxymethylene-2-phenyl-4*H*-oxazol-5-one (Matos *et al.*, 2003) (0.01 mol) and 3-methoxy-phenylamine (0.01 mol) in THF (25 ml) was stirred at room temperature for 4 h. The solvent was then evaporated under reduced pressure and the residue was crystallized from ethyl acetate to give orange crystals suitable for X-ray analysis (yield 82%).

### Refinement

Amino-H atom was located in a difference Fourier map and refined isotropically. Other H atoms were placed in calculated positions and refined as riding atoms with C—H = 0.95–0.98 Å, *U*_iso_(H) = 1.2–1.5*U*_eq_(C).

### Crystal data

**Table:**

C_17_H_14_N_2_O_3_	*Z* = 2
*M**_r_* = 294.30	*F*(000) = 308
Triclinic, *P*1	*D*_x_ = 1.442 Mg m^−^^3^
Hall symbol: -P 1	Mo *K*α radiation, λ = 0.71073 Å
*a* = 6.6085 (5) Å	Cell parameters from 2524 reflections
*b* = 7.1887 (5) Å	θ = 3.0–28.7°
*c* = 15.3659 (10) Å	µ = 0.10 mm^−^^1^
α = 98.629 (5)°	*T* = 150 K
β = 94.096 (5)°	Block, green
γ = 108.715 (6)°	0.25 × 0.20 × 0.15 mm
*V* = 677.96 (8) Å^3^	

### Data collection

**Table:**

Agilent SuperNova (single source at offset) Eos diffractometer	2759 independent reflections
Radiation source: fine-focus sealed tube	2317 reflections with *I* > 2σ(*I*)
Graphite monochromator	*R*_int_ = 0.023
ω scans	θ_max_ = 26.4°, θ_min_ = 3.0°
Absorption correction: multi-scan (*CrysAlis PRO*; Agilent, 2011)	*h* = −8→8
*T*_min_ = 0.975, *T*_max_ = 0.985	*k* = −8→8
5557 measured reflections	*l* = −19→19

### Refinement

**Table:**

Refinement on *F*^2^	Primary atom site location: structure-invariant direct methods
Least-squares matrix: full	Secondary atom site location: difference Fourier map
*R*[*F*^2^ > 2σ(*F*^2^)] = 0.043	Hydrogen site location: inferred from neighbouring sites
*wR*(*F*^2^) = 0.118	H atoms treated by a mixture of independent and constrained refinement
*S* = 1.02	*w* = 1/[σ^2^(*F*_o_^2^) + (0.060*P*)^2^ + 0.1704*P*] where *P* = (*F*_o_^2^ + 2*F*_c_^2^)/3
2759 reflections	(Δ/σ)_max_ < 0.001
204 parameters	Δρ_max_ = 0.22 e Å^−^^3^
0 restraints	Δρ_min_ = −0.29 e Å^−^^3^

### Special details

**Table:**

Experimental. ^1^H NMR (DMSO, 500 MHz) *δ*: 10.70 (d, 1H, NH), 7.97–8.05 (m, 3H, Ar—H), 7.55–7.57(m, 3H, Ar—H), 7.25 (t, 1H, Ar—H), 7.10–7.14 (m, 2H, Ar—H), 6.67 (q, 1H, Ar—H), 3.78 (s, 3H, CH3). ^13^C NMR (DMSO, 125.77 MHz) *δ*: 167.11, 160.27, 154.44, 141.21, 135.31, 131.34, 130.28, 129.09, 126.55, 126.40, 125.82, 111.06, 109.81, 109.29, 102.70, 55.23.
Geometry. All e.s.d.'s (except the e.s.d. in the dihedral angle between two l.s. planes) are estimated using the full covariance matrix. The cell e.s.d.'s are taken into account individually in the estimation of e.s.d.'s in distances, angles and torsion angles; correlations between e.s.d.'s in cell parameters are only used when they are defined by crystal symmetry. An approximate (isotropic) treatment of cell e.s.d.'s is used for estimating e.s.d.'s involving l.s. planes.
Refinement. Refinement of *F*^2^ against ALL reflections. The weighted *R*-factor *wR* and goodness of fit *S* are based on *F*^2^, conventional *R*-factors *R* are based on *F*, with *F* set to zero for negative *F*^2^. The threshold expression of *F*^2^ > σ(*F*^2^) is used only for calculating *R*-factors(gt) *etc*. and is not relevant to the choice of reflections for refinement. *R*-factors based on *F*^2^ are statistically about twice as large as those based on *F*, and *R*- factors based on ALL data will be even larger.

### Fractional atomic coordinates and isotropic or equivalent isotropic displacement parameters (Å^2^)

**Table:**

	*x*	*y*	*z*	*U*_iso_*/*U*_eq_	
C1	0.2537 (3)	0.6133 (3)	0.85604 (11)	0.0242 (4)	
H1	0.3396	0.5732	0.8151	0.029*	
C2	0.3478 (3)	0.7213 (3)	0.93963 (11)	0.0311 (4)	
H2	0.4981	0.7544	0.9562	0.037*	
C3	0.2225 (3)	0.7811 (3)	0.99922 (11)	0.0318 (4)	
H3	0.2876	0.8552	1.0565	0.038*	
C4	0.0043 (3)	0.7337 (3)	0.97570 (11)	0.0297 (4)	
H4	−0.0803	0.7762	1.0165	0.036*	
C5	−0.0917 (3)	0.6238 (3)	0.89242 (11)	0.0243 (4)	
H5	−0.2425	0.5895	0.8766	0.029*	
C6	0.0327 (2)	0.5636 (2)	0.83184 (10)	0.0189 (3)	
C7	−0.0642 (2)	0.4518 (2)	0.74310 (10)	0.0176 (3)	
C8	−0.3384 (3)	0.2824 (2)	0.63810 (10)	0.0192 (3)	
C9	−0.1337 (2)	0.3036 (2)	0.60730 (10)	0.0177 (3)	
C10	−0.1006 (2)	0.2327 (2)	0.52347 (10)	0.0177 (3)	
H10	−0.2216	0.1660	0.4796	0.021*	
C11	0.1565 (3)	0.2043 (2)	0.41664 (9)	0.0179 (3)	
C12	0.3751 (3)	0.2674 (2)	0.40875 (10)	0.0194 (3)	
H12	0.4774	0.3409	0.4588	0.023*	
C13	0.4433 (3)	0.2222 (2)	0.32722 (10)	0.0214 (4)	
H13	0.5925	0.2669	0.3213	0.026*	
C14	0.2950 (3)	0.1127 (2)	0.25468 (10)	0.0220 (4)	
H14	0.3422	0.0818	0.1991	0.026*	
C15	0.0758 (3)	0.0477 (2)	0.26330 (10)	0.0184 (3)	
C16	0.0038 (2)	0.0943 (2)	0.34428 (10)	0.0184 (3)	
H16	−0.1457	0.0522	0.3499	0.022*	
C17	−0.2821 (3)	−0.1308 (3)	0.19348 (11)	0.0292 (4)	
H17A	−0.3122	−0.2117	0.2400	0.044*	
H17B	−0.3592	−0.2118	0.1364	0.044*	
H17C	−0.3300	−0.0154	0.2077	0.044*	
N1	0.0328 (2)	0.41031 (19)	0.67659 (8)	0.0183 (3)	
N2	0.0970 (2)	0.2548 (2)	0.50167 (9)	0.0194 (3)	
O1	−0.52599 (17)	0.20389 (18)	0.60520 (7)	0.0254 (3)	
O2	−0.28635 (16)	0.38163 (16)	0.72695 (7)	0.0195 (3)	
O3	−0.05667 (17)	−0.06328 (17)	0.18811 (7)	0.0235 (3)	
H2A	0.211 (3)	0.304 (3)	0.5461 (13)	0.036 (5)*	

### Atomic displacement parameters (Å^2^)

**Table:**

	*U*^11^	*U*^22^	*U*^33^	*U*^12^	*U*^13^	*U*^23^
C1	0.0235 (9)	0.0242 (9)	0.0223 (8)	0.0044 (7)	0.0045 (7)	0.0031 (7)
C2	0.0262 (9)	0.0315 (10)	0.0271 (9)	0.0013 (8)	−0.0029 (7)	0.0013 (8)
C3	0.0432 (11)	0.0260 (10)	0.0179 (8)	0.0048 (8)	−0.0032 (8)	−0.0026 (7)
C4	0.0415 (11)	0.0292 (10)	0.0195 (8)	0.0148 (8)	0.0068 (8)	0.0006 (7)
C5	0.0278 (9)	0.0256 (9)	0.0211 (8)	0.0117 (7)	0.0037 (7)	0.0033 (7)
C6	0.0229 (8)	0.0160 (8)	0.0172 (8)	0.0054 (6)	0.0026 (6)	0.0043 (6)
C7	0.0183 (8)	0.0166 (8)	0.0189 (8)	0.0063 (6)	0.0037 (6)	0.0046 (6)
C8	0.0226 (9)	0.0213 (8)	0.0153 (7)	0.0096 (7)	0.0034 (6)	0.0030 (6)
C9	0.0182 (8)	0.0186 (8)	0.0167 (7)	0.0066 (6)	0.0029 (6)	0.0034 (6)
C10	0.0190 (8)	0.0176 (8)	0.0174 (7)	0.0074 (6)	0.0016 (6)	0.0031 (6)
C11	0.0244 (8)	0.0161 (8)	0.0161 (8)	0.0098 (6)	0.0053 (6)	0.0038 (6)
C12	0.0200 (8)	0.0185 (8)	0.0188 (8)	0.0061 (6)	0.0022 (6)	0.0018 (6)
C13	0.0199 (8)	0.0222 (9)	0.0233 (8)	0.0076 (7)	0.0070 (7)	0.0047 (7)
C14	0.0247 (9)	0.0258 (9)	0.0178 (8)	0.0106 (7)	0.0086 (7)	0.0035 (7)
C15	0.0225 (8)	0.0172 (8)	0.0163 (7)	0.0075 (6)	0.0022 (6)	0.0034 (6)
C16	0.0176 (8)	0.0200 (8)	0.0201 (8)	0.0085 (6)	0.0056 (6)	0.0052 (6)
C17	0.0196 (9)	0.0384 (11)	0.0251 (9)	0.0087 (8)	0.0001 (7)	−0.0043 (7)
N1	0.0184 (7)	0.0196 (7)	0.0170 (6)	0.0064 (5)	0.0037 (5)	0.0028 (5)
N2	0.0170 (7)	0.0246 (8)	0.0156 (7)	0.0073 (6)	0.0025 (5)	0.0001 (5)
O1	0.0177 (6)	0.0362 (7)	0.0209 (6)	0.0083 (5)	0.0005 (5)	0.0030 (5)
O2	0.0157 (6)	0.0264 (6)	0.0151 (5)	0.0068 (5)	0.0031 (4)	−0.0003 (4)
O3	0.0217 (6)	0.0296 (7)	0.0158 (6)	0.0059 (5)	0.0027 (5)	−0.0002 (5)

### Geometric parameters (Å, º)

**Table:**

C1—C2	1.384 (2)	C10—N2	1.337 (2)
C1—C6	1.395 (2)	C10—H10	0.9500
C1—H1	0.9500	C11—C12	1.389 (2)
C2—C3	1.388 (3)	C11—C16	1.394 (2)
C2—H2	0.9500	C11—N2	1.4201 (19)
C3—C4	1.378 (3)	C12—C13	1.389 (2)
C3—H3	0.9500	C12—H12	0.9500
C4—C5	1.387 (2)	C13—C14	1.381 (2)
C4—H4	0.9500	C13—H13	0.9500
C5—C6	1.396 (2)	C14—C15	1.395 (2)
C5—H5	0.9500	C14—H14	0.9500
C6—C7	1.460 (2)	C15—O3	1.3670 (19)
C7—N1	1.2917 (19)	C15—C16	1.396 (2)
C7—O2	1.3810 (18)	C16—H16	0.9500
C8—O1	1.2191 (19)	C17—O3	1.4237 (19)
C8—O2	1.4051 (18)	C17—H17A	0.9800
C8—C9	1.435 (2)	C17—H17B	0.9800
C9—C10	1.372 (2)	C17—H17C	0.9800
C9—N1	1.407 (2)	N2—H2A	0.92 (2)
			
C2—C1—C6	120.00 (16)	C12—C11—C16	121.14 (14)
C2—C1—H1	120.0	C12—C11—N2	116.95 (14)
C6—C1—H1	120.0	C16—C11—N2	121.90 (14)
C1—C2—C3	120.03 (17)	C13—C12—C11	119.60 (15)
C1—C2—H2	120.0	C13—C12—H12	120.2
C3—C2—H2	120.0	C11—C12—H12	120.2
C4—C3—C2	120.38 (16)	C14—C13—C12	120.30 (15)
C4—C3—H3	119.8	C14—C13—H13	119.8
C2—C3—H3	119.8	C12—C13—H13	119.8
C3—C4—C5	120.01 (16)	C13—C14—C15	119.82 (14)
C3—C4—H4	120.0	C13—C14—H14	120.1
C5—C4—H4	120.0	C15—C14—H14	120.1
C4—C5—C6	120.10 (16)	O3—C15—C14	115.12 (13)
C4—C5—H5	119.9	O3—C15—C16	124.11 (14)
C6—C5—H5	119.9	C14—C15—C16	120.77 (15)
C1—C6—C5	119.48 (15)	C11—C16—C15	118.35 (14)
C1—C6—C7	119.49 (14)	C11—C16—H16	120.8
C5—C6—C7	121.03 (15)	C15—C16—H16	120.8
N1—C7—O2	115.14 (13)	O3—C17—H17A	109.5
N1—C7—C6	127.89 (14)	O3—C17—H17B	109.5
O2—C7—C6	116.97 (13)	H17A—C17—H17B	109.5
O1—C8—O2	120.54 (14)	O3—C17—H17C	109.5
O1—C8—C9	135.12 (15)	H17A—C17—H17C	109.5
O2—C8—C9	104.33 (13)	H17B—C17—H17C	109.5
C10—C9—N1	124.09 (14)	C7—N1—C9	104.91 (13)
C10—C9—C8	126.24 (15)	C10—N2—C11	128.04 (14)
N1—C9—C8	109.67 (13)	C10—N2—H2A	118.3 (12)
N2—C10—C9	121.89 (15)	C11—N2—H2A	113.6 (13)
N2—C10—H10	119.1	C7—O2—C8	105.95 (11)
C9—C10—H10	119.1	C15—O3—C17	117.09 (12)
			
C6—C1—C2—C3	−0.4 (3)	C12—C13—C14—C15	0.2 (2)
C1—C2—C3—C4	0.0 (3)	C13—C14—C15—O3	−178.56 (13)
C2—C3—C4—C5	0.6 (3)	C13—C14—C15—C16	0.9 (2)
C3—C4—C5—C6	−0.9 (3)	C12—C11—C16—C15	0.4 (2)
C2—C1—C6—C5	0.1 (2)	N2—C11—C16—C15	−178.82 (13)
C2—C1—C6—C7	179.27 (15)	O3—C15—C16—C11	178.24 (13)
C4—C5—C6—C1	0.5 (2)	C14—C15—C16—C11	−1.2 (2)
C4—C5—C6—C7	−178.59 (14)	O2—C7—N1—C9	0.53 (17)
C1—C6—C7—N1	−9.5 (2)	C6—C7—N1—C9	−179.07 (14)
C5—C6—C7—N1	169.59 (15)	C10—C9—N1—C7	179.11 (14)
C1—C6—C7—O2	170.88 (13)	C8—C9—N1—C7	−0.69 (16)
C5—C6—C7—O2	−10.0 (2)	C9—C10—N2—C11	−174.39 (14)
O1—C8—C9—C10	0.7 (3)	C12—C11—N2—C10	171.24 (15)
O2—C8—C9—C10	−179.20 (14)	C16—C11—N2—C10	−9.5 (2)
O1—C8—C9—N1	−179.47 (17)	N1—C7—O2—C8	−0.17 (17)
O2—C8—C9—N1	0.59 (16)	C6—C7—O2—C8	179.48 (12)
N1—C9—C10—N2	2.3 (2)	O1—C8—O2—C7	179.78 (14)
C8—C9—C10—N2	−177.99 (14)	C9—C8—O2—C7	−0.27 (15)
C16—C11—C12—C13	0.7 (2)	C14—C15—O3—C17	−179.23 (14)
N2—C11—C12—C13	179.92 (14)	C16—C15—O3—C17	1.3 (2)
C11—C12—C13—C14	−1.0 (2)		

### Hydrogen-bond geometry (Å, º)

**Table:**

*D*—H···*A*	*D*—H	H···*A*	*D*···*A*	*D*—H···*A*
N2—H2*A*···O1^i^	0.92 (2)	2.26 (2)	3.0110 (18)	138.1 (17)

Symmetry code: (i) *x*+1, *y*, *z*.
